# Expedient Synthesis of Lupulones and Their Derivatization to 2,8‐7*H*‐Dihydrochromen‐7‐ones

**DOI:** 10.1002/open.202000008

**Published:** 2020-04-06

**Authors:** Lena Decuyper, Gurkirat Kaur, Charlotte Versyck, Eline Blondeel, Yves Depetter, Kristof Van Hecke, Matthias D'hooghe

**Affiliations:** ^1^ Department of Green Chemistry and Technology Faculty of Bioscience Engineering Ghent University Coupure Links 653 9000 Ghent Belgium; ^2^ Department of Chemistry Faculty of Sciences Ghent University Krijgslaan 281-S3 9000 Ghent Belgium

**Keywords:** Lupulones, chromenones, hop and beer bitter acids, triprenylation, medicinal chemistry

## Abstract

A convenient and improved method for the synthesis of beta acids or lupulones, which are known to possess *e. g*. anti‐cancer, anti‐inflammatory, anti‐oxidative and antimicrobial activity, has been developed successfully. Further derivatization of these complex structures to the corresponding dihydrochromen‐7‐ones, including the natural product machuone, was realized to simplify their analysis and to confirm their molecular structure. In addition to practical and safe laboratory procedures, the advantages associated with this new approach involve the use of water as a solvent and the direct crystallization of lupunones from acetonitrile, rendering our strategy more efficient and benign as compared to available methods.

## Graphical abstract

**Don't worry, be hoppy**! This Communication reports on the development of a convenient and improved method for the synthesis of biologically relevant lupulones, which underwent further derivatization to the corresponding dihydrochromen‐7‐ones. This new approach is more practical, efficient and benign as compared to available strategies because of *e. g*. the use of water as a solvent and the direct crystallization of lupunones from acetonitrile.

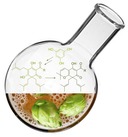

The hop plant (*Humulus lupulus*) has been inextricably linked to beer thanks to the contribution of hop to the typical bitter taste and the aroma.^1^ Moreover, hop also exerts a positive effect on the stability of the beer foam.^2^ These effects are, *inter alia*, assigned to the so‐called hop acids present in the lupulin glands of hop cones. Beta acids or lupulones **2** are natural products originating from the lupulin glands on female inflorescences of the hop plant.^1^ However, alpha acids or humulones **1** are often considered as the most important metabolites in hop as they significantly add to the typical bitter taste and the unique aroma of beer (Figure 1). Consequently, these compounds received considerable attention from a scientific point of view, including various studies related to their chemical synthesis and biological activities. Nonetheless, although beta acids as such play a minor role in beer brewery, these structures do act as precursors of valuable compounds important for beer quality. Beta acids and their derivatives are also relevant from a medicinal point of view, as they have been shown to display antimicrobial activity toward Gram‐positive bacteria and fungi, and they are known to demonstrate anticancer, anti‐inflammatory and anti‐oxidative properties as well.^3^

**Figure 1:**
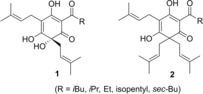
Hop or bitter acids.

Bearing this in mind, hop acids can be considered as eligible substrates for the development of new biologically active compounds. Extraction of bitter acids from the hop plant is, however, not straightforward. This process is time and labour intensive and requires harsh conditions such as high temperatures and pressures, suggesting the need for a chemical alternative.^4^ On the other hand, the available chemical syntheses suffer from limitations such as lack of selectivity, practical and safety issues and low yields.

In summary, lupulones (i) received considerably less attention in the literature as compared to their humulone counterparts and (ii) constitute interesting substrates for bioactive compound development, but (iii) they are difficult to obtain due to cumbersome isolation or inefficient synthesis procedures. Consequently, the main objective of this work involved the development of a convenient and practical alternative method for lupulone synthesis and their structural investigation.

The premised synthesis of lupulones commenced with monoacylation of phloroglucinol **3** by means of a traditional Friedel‐Crafts acylation protocol, relying on a classical Fries rearrangement (Scheme 1).^5^ To that end, phloroglucinol **3** was dissolved in nitrobenzene under inert atmosphere (Ar), after which acylation took place deploying various acid chlorides at high temperature mediated by aluminium(III) chloride, giving rise to acylphloroglucinols **4 a**–**e**. After purification *via* column chromatography (SiO_2_) and, if necessary, crystallization from water/acetonitrile (1 : 1), pure aryl ketones **4 a**–**e** were obtained and subsequently transformed into the envisioned lupulones **5 a**–**e** *via* a triple prenylation protocol.

**Scheme 1:**
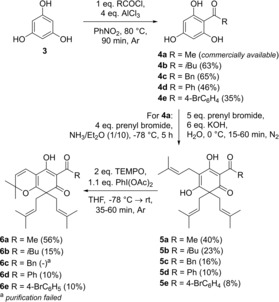
Synthesis of lupulones **5 a**–**e** and 2,8‐dihydro‐7*H*‐chromen‐7‐one derivatives **6 a**–**e**.

The desired triprenylation of compounds **4 a**–**e** was effected by three consecutive base‐mediated electrophilic substitution reactions with prenyl bromide. It should be noted, though, that the occurrence of mono‐, di‐ and tetraprenylated side products is known to present a significant hurdle in that respect. We first tested the procedure reported by Drewett and Laws, the most frequently used and highest yielding method for the synthesis of analogous lupulones available in the literature.^6^ To that end, commercially available acetophloroglucinol monohydrate **4 a** was dissolved in dry diethyl ether and added to dry, liquid ammonia at −78 °C. The reaction was then started by the addition of four equivalents of prenyl bromide (instead of the reported 7.5 eq). After reaction and work‐up as described,^3^ acetolupulone **5 a** was obtained in 35 % yield. However, due to the hygroscopic character of ammonia, its tendency to react with CO_2_, its low boiling point and high explosion risk, the latter reaction protocol is not devoid of risks.^7^

To search for a more efficient, safe and practical method for triprenylation of acylphloroglucinols **4 a**–**e**, a patented procedure based on the use of the tertiary alcohol 2‐methylbut‐3‐en‐2‐ol and zinc(II) chloride as a Lewis acid catalyst was tested next.^8^ However, we were soon urged to look for an alternative based on irreproducible results and the formation of multiple side products.

Next, reaction conditions reported by George *et al*. were tested and optimized, which had been applied for the synthesis of *m*‐diprenylacylphloroglucinol **8**, a deoxyhumulone, but gave *gem*‐diprenylated acylphloroglucinol **9** and triprenylated lupulone **10** as side products (Scheme 2).^9^

**Scheme 2:**
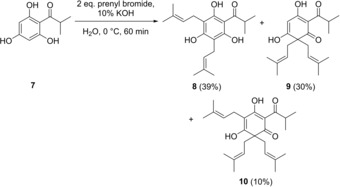
Synthesis of deoxyhumulone **8** by George *et al*.^9^

Our attempts to adapt George's reaction parameters (Scheme 2) to increase the selectivity toward formation of lupulones **5** at the expense of mono‐, di‐ or tetraprenylated side products are summarized in Table 1. To that end, compound **4 a** was dissolved in ice‐cooled water along with potassium hydroxide and prenyl bromide. The reaction time, concentration and numbers of equivalents of the reagents were varied to evaluate the conversion rate and the formation of side products during the reaction. Apparently, the combination of five and six equivalents of prenyl bromide and KOH (35.7 g/L), respectively, proved to be favorable and afforded lupulone **5 a** in 40 % yield after crystallization from acetonitrile (entry 7). As comprehensive characterization of lupulone **5 a** on the basis of NMR spectral data was complicated due to the presence of several tautomeric forms, the structure was additionally secured by means of X‐ray crystallography (Figure 2). Next, our new procedure was validated through the successful synthesis of other alkyl‐substituted analogues, natural product *n*‐lupulone **5 b** and phenylacetolupulone **5 c** in an identical way, although slow, cooled crystallization and recrystallization from acetonitrile were necessary to obtain pure compounds, resulting in slightly lower yields for these derivatives. For aryl analogues **5 d**–**e**, the reaction time was limited to 15 minutes as at this point, the percentages of trisubstituted lupulones **5 d**–**e** already reached their maximal level (>86 %), while the amounts of side products were kept at a minimal degree (<4 % diprenylated and <8 % tetraprenylated products). Purification by means of reversed‐phase column chromatography eventually furnished pure target compounds **5 d**–**e**.

**Table 1:** Optimization of the prenylation of **4 a**.

Entry	Eq. prenyl bromide	Eq. KOH	Conc. KOH [g/L]	Time [h]	Yield [%] after crystallization from CH_3_CN
1	3	3	26.6	1	5
2	4	3	26.6	1	8
3	5	3	26.6	1	11
4	5	6	44.3	1	17
5	5	6	44.3	3	18
6	5	6	56.7	1	14
**7**	**5**	**6**	**35.7**	**1**	**40**
8	5	6	35.7	3	26

**Figure 2:**
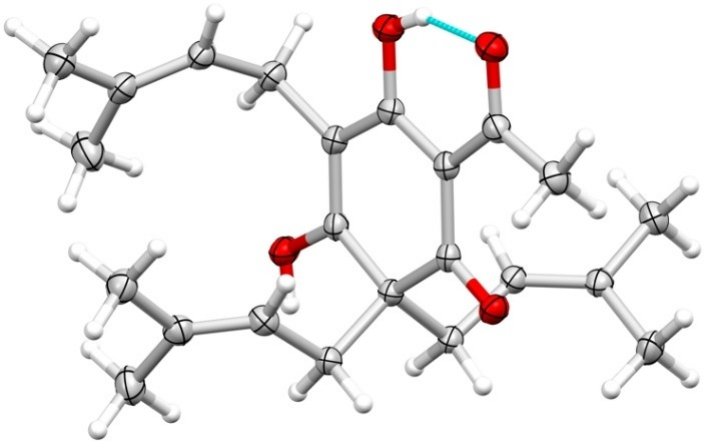
Molecular X‐ray structure of acetolupulone **5 a**, thermal displacement ellipsoids are shown at the 50 % probability level. An intramolecular hydrogen bond is indicated.

As such, based on the higher yield obtained (for **5 a**) and its less time‐consuming nature, our new method represents a suitable alternative to the method of Drewett and Laws.^3^ In particular, it is considerably more practical and safe, time efficient, uses water as a solvent, and allows for direct crystallization from acetonitrile for alkyl derivatives.

As mentioned above, comprehensive characterization of lupulones **5** on the basis of NMR spectral data was seriously complicated by the presence of multiple tautomeric forms. Besides X‐ray analysis for compound **5 a**, further elucidation and confirmation of the structural identity of the synthesized, in some cases non‐crystalline, products **5** was therefore enabled through their oxidative intramolecular cyclization into 2,8‐dihydro‐7*H*‐chromen‐7‐one derivatives, in accordance with a literature protocol (Scheme 1).^9^ Thus, lupulones **5** were treated with 2,2,6,6‐tetramethylpiperidinyloxyl (TEMPO) and (diacetoxyiodo)‐benzene in dry THF at −78 °C, initiating a 6π‐electrocyclic reaction of the *O*‐quinone methide intermediate, formed through selective hydride withdrawal from precursor **5** by the *in situ* generated TEMPO cation.^5^ Purification *via* column chromatography (SiO_2_ or C18) and NMR analysis of the compounds produced in this way eventually confirmed the structure of cyclic derivatives **6** (as a mixture of two tautomeric forms for **6 a**–**b**) and hence their acyclic counterparts **5**. The polyisoprenylated benzophenone machuone **6 d** has previously been isolated from the fruits of *Clusia columnaris* and *Clusia sandiensis*, two plants found in tropic and subtropic areas in Central and South America,^10^ pointing to the relevance of these chromenone structures from a natural product chemistry point of view as well.

In conclusion, a practical and convenient new approach toward the synthesis of lupulone‐like structures has been accomplished, allowing for the preparation of a variety of beta acid analogues in the framework of *e. g*. medicinal chemistry studies. Furthermore, cyclization of these lupulones expectedly delivered dihydrochromen‐7‐one derivatives, allowing for an easier structural analysis. Moreover, by encapsulation of the isolated prenyl group in the chromenone fused ring, it is also conceivable that these natural product‐based structures are more resistant toward oxidative decomposition when compared to their lupulone precursors, which are typically easily degraded to bitter hulupones.

## Conflict of interest

The authors declare no conflict of interest.

## Supporting information

As a service to our authors and readers, this journal provides supporting information supplied by the authors. Such materials are peer reviewed and may be re‐organized for online delivery, but are not copy‐edited or typeset. Technical support issues arising from supporting information (other than missing files) should be addressed to the authors.

Supplementary
