# Supplementary material for: Expedient Synthesis of Lupulones and Their Derivatization to 2,8‐7H‐Dihydrochromen‐7‐ones
Source: ChemistryOpen. 2020 Apr 6;9(4):442–4. doi: 10.1002/open.202000008 (PMC7136647; doi:10.1002/open.202000008)
Supplement: Supplementary file 1 — Supplementary [file OPEN-9-442-s001.pdf]

ChemistryOpen

Supporting Information

Expedient Synthesis of Lupulones and Their Derivatization to 2,8-7*H*-Dihydrochromen-7-ones

Lena Decuyper, Gurkirat Kaur, Charlotte Versyck, Eline Blondeel, Yves Depetter, Kristof Van Hecke, and Matthias D'hooghe*© 2020 The Authors. Published by Wiley-VCH Verlag GmbH & Co. KGaA. This is an open access article under the terms of the Creative Commons Attribution License, which permits use, distribution and reproduction in any medium, provided the original work is properly cited.

SUPPORTING INFORMATION

1 General methods

Dry THF was obtained using the MBraun SPS-800 solvent purification system. Thin layer chromatography (TLC) analysis of reaction mixtures or pure samples was performed using glass-backed 0.25-mm Merck silica gel 60 F_{254} TLC plates, and visualized under UV light (254 nm) or by using a $KMnO_4$ stain. Column chromatography was carried out on chromatographic silica gel (particle size 35-70 µm, pore diameter 6 nm). Automated preparative HPLC separations were executed with an Agilent 1100 Series apparatus with a UV detector, using a Supelco Ascentis® C18 column (I.D. x L 21.1 mm x 150 mm), characterized by a 5 µm particle size. The eluent mixtures consisted of water and acetonitrile. Automated column chromatography was performed on a Büchi Reveleris® X2 flash chromatography system (normal phase) or Grace Reveleris® X1 flash chromatography system (reversed phase), using prepacked Reveleris® silica or Reveleris® C18 cartridges.

^{1}H NMR and ^{13}C NMR spectra were recorded at 25 °C at 400 and 100 MHz, respectively, using a Bruker Avance III-400 spectrophotometer, equipped with ^{1}H/BB z-gradient probe (BBO, 5 mm). The samples were dissolved in deuterated solvents (with tetramethylsilane as internal standard). All spectra were processed using TOPSPIN 3.2 and acquired through the standard sequences available in the Bruker pulse programme library. ^{1}H and ^{13}C chemical shifts (δ) are reported in parts per million (ppm) downfield of TMS and referenced to the residual solvent peak ($CDCl_3$ δ_H = 7.26, δ_C = 77.16; $(CD_3)_2SO$ δ_H = 2.50, δ_C = 39.52). Coupling constants (*J*) are reported in hertz (Hz). Peaks were assigned with the aid of 2D spectra (COSY, HSQC, HMBC). IR spectra were obtained from samples in neat form with an ATR (Attenuated Total Reflectance) accessory on a PerkinElmer Spectrum BX FT-IR or Shimadzu IRAffinity-1S WL FT-IR spectrophotometer. Only selected absorbances (ν_{max}, cm^{-1}) are reported. HPLC and HPLC-MS analyses were performed on an Agilent 1200 series HPLC system fitted with an Ascentis® Express C18 column (particle size 2.7 µm, length 30 mm, internal diameter 4.6 mm) and connected to a UV-VIS detector and an Agilent 1100 series LC/MSD-type SL mass spectrometer (ESI, 70 eV) using a mass-selective single-quadrupole detector. A mixture of acetonitrile/water (5 mM NH_4OAc) was used as the eluent. Automated preparative HPLC separations were executed with an Agilent 1100 Series apparatus with a UV detector, using a Zorbax® Eclipse XDB-C18 column (I.D. x L 21.2 mm x 150 mm), characterized by a 5 µm particle size. The eluent mixtures consisted of water and acetonitrile. Melting points were measured using a Kofler heating bench system of Wagner & Munz (type WME, accuracy ± 1 °C).

2 Single crystal X-ray diffraction

X-ray analysis was performed by Prof. Kristof Van Hecke (XStruct, Department of Chemistry, Faculty of Sciences, Ghent University). For the structure of compound **5a**, X-ray intensity data were collected at 100 K on a Rigaku Oxford Diffraction Supernova Dual Source (Cu at zero) diffractometer equipped with an Atlas CCD detector using ω scans and CuKα (λ = 1.54184 Å) radiation. The images were interpreted and integrated with the program

CrysAlisPro [i]. Using Olex2 [ii], the structure was solved by direct methods using the ShelXS structure solution program and refined by full-matrix least-squares on F2 using the ShelXL program package [iii, iv]. Non-hydrogen atoms were anisotropically refined and the hydrogen atoms in the riding mode and isotropic temperature factors fixed at 1.2 times U(eq) of the parent atoms (1.5 times for methyl and hydroxyl groups).

CCDC 1977252 contains the supplementary crystallographic data for these molecules and can be obtained free of charge via www.ccdc.cam.ac.uk/conts/retrieving.html (or from the Cambridge Crystallographic Data Centre, 12, Union Road, Cambridge CB2 1EZ, UK; fax: +44-1223-336033; or deposit@ccdc.cam.ac.uk).

[i] Rigaku Oxford Diffraction (2015). CrysAlis Pro; Rigaku Oxford Diffraction, Yarnton, England.

[ii] O.V. Dolomanov, L.J. Bourhis, R.J. Gildea, J.A.K. Howard, H. Puschmann, J. Appl. Crystallogr. 42 (2009) 339-341

[iii] G.M. Sheldrick, Acta Crystallogr. Sect. A64 (2008) 112-122.

[iv] G.M. Sheldrick, Acta Crystallogr. Sect. C71 (2015) 3-8.

3 Synthesis of acylphloroglucinols 4a-e

The synthesis of 2,4,6-trihydroxyisovalerophenone **4b** serves as an example for the synthesis of compounds **4a-e**. In a flame-dried flask of 50 mL, 1 g (8 mmol) phloroglucinol **3** was dissolved in 15 mL nitrobenzene, along with 4.22 g (32 mmol; 4 eq.) aluminium(III) chloride and stirred at room temperature for 30 minutes under argon atmosphere. Next, 0.97 mL (8 mmol; 1 eq.) isovaleryl chloride was added dropwise, after which the reaction mixture was heated at 80 °C for 90 minutes. To quench the reaction, the flask was cooled to 0 °C by means of an ice bath, and 10 mL of HCl (aq., 3 M) and 15 mL of distilled water were carefully added. The aqueous mixture was extracted three times with 30 mL EtOAc, after which the combined organic phases were washed with saturated aq. $NaHCO_3$ (2 x 30 mL), distilled water (2 x 30 mL) and brine (30 mL). The solution was then dried over magnesium sulfate, filtered and the solvent evaporated *in vacuo*. Purification was performed using column chromatography (SiO_2, petroleum ether/ethyl acetate (3/1), R_f = 0.18) and furnished a yellow oil. The oil was redissolved in 10 mL water/acetonitrile (1/1). After evaporation of the solvent, 1.05 g (4.98 mmol, 63%) 2,4,6-trihydroxyisvalerophenone **4b** was obtained in crystalline form. For the purification of compounds **4c** and **4d**, a 2/1-mixutre of petroleum ether/EtOAc was used as the eluent, for compound **4e**, a 3/2-mixture was used.

The spectral data of compounds **4b**, **4c** and **4d** are in accordance with those found in the literature.[1,2,3,4]

(4-Bromophenyl)(2,4,6-trihydroxyphenyl)methanone 4e

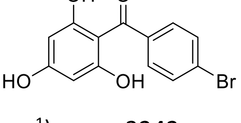

^{1}H NMR (400 MHz, δ_6-DMSO): δ 5.85 (2H, s); 7.54 (2H, d, *J* = 8.5 Hz); 7.65 (2H, d, *J* = 8.5 Hz); 9.90 (1H, s); 10.17 (2H, s). **^{13}C NMR** (100.6 MHz, δ_6-DMSO): δ 94.9 (CH); 105.7 (C_q); 125.9 (C_q); 130.9 (CH); 131.5 (CH); 139.5 (C_q); 160.0 (C_q); 162.7 (C_q); 195.8 (C=O). **IR** (ATR, cm^{-1}): ν_{OH} = 3242; $\nu_{C=O}$ = 1639; ν_{max} = 1585, 1151, 1061, 1013, 925, 520. **MS** (70 eV): m/z (%) 309/11 (M^+ + 1, 80); 203 (100). Yellow crystals. T_m = 210 °C. Yield after column chromatography: 26%. R_f = 0.15 (SiO_2, PE/EtOAc 3/2).

4 Synthesis of lupulones 5a-e

The synthesis of acetolupulone **5a** serves as an example for the synthesis of lupulones **5a-e**. In a flask of 100 mL, 1 g 2,4,6-trihydroxyacetophenone monohydrate **4a** (5 mmol) and 1.77 g KOH (30 mmol; 6 eq.) were dissolved in 50 mL water and cooled to 0 °C by means of an ice bath. To this mixture, 3.25 mL prenyl bromide (25 mmol, 5 eq.) was slowly added dropwise. The reaction mixture was stirred for one hour under nitrogen atmosphere (15 minutes for aryl analogues **4d-e**). Next, 1 mL of HCl (3 M) was added. The mixture was extracted with EtOAc (3 x 50 mL) and the organic phase washed with a saturated solution of NaCl, dried with $MgSO_4$, filtered and evaporated *in vacuo*. Crystallization in acetonitrile furnished 809 mg (2.18 mmol; 40%) acetolupulone **5a**.

For the synthesis of compounds **5b-c**, recrystallization in acetonitrile was necessary to remove impurities. Compounds **5d-e** were purified by means of reversed phase column chromatography.

The spectral data of compounds **5a-c** are in accordance with literature data.[4,5] Comprehensive characterization of lupulones **5d-e** on the basis of NMR spectral data was complicated by the presence of several tautomeric forms, but LC-MS analysis did give a correct indication regarding the identity and purity of these new compounds.

2-Benzoyl-3,5-dihydroxy-4,6,6-tris(3-methylbut-2-en-1-yl)cyclohexa-2,4-dien-1-one 5d

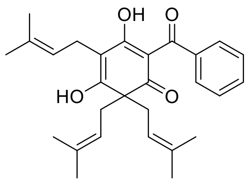

MS (70 eV): m/z (%) 435 (M^+ + 1, 100).

2-(4-Bromobenzoyl)-3,5-dihydroxy-4,6,6-tris(3-methylbut-2-en-1-yl)cyclohexa-2,4-dien-1-one 5e

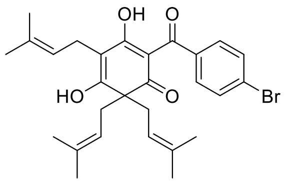

MS (70 eV): m/z (%) 513/15 (M^+ + 1, 100).

5 Synthesis of 6-acyl-5-hydroxy-2,2-dimethyl-8,8-bis(3-methylbut-2-en-1-yl)-2,8-dihydro-7*H*-chromen-7-ones 6a-e

The synthesis of 6-acetyl-5-hydroxy-2,2-dimethyl-8,8-bis(3-methylbut-2-en-1-yl)-2,8-dihydro-7*H*-chromen-7-one **6a** serves as an example for the synthesis of compounds **6a-e**. In a flame-dried flask of 25 mL, 200 mg (0.5 mmol) acetolupulone **5a** was dissolved in 10 mL dry THF, put under argon atmosphere and cooled to -78 °C. 171 mg (1 mmol, 2 eq.) TEMPO and 190 mg (0.55 mmol, 1.1 eq.) $PhI(OAc)_2$ were added. After five minutes, the reaction mixture was allowed to warm up to room temperature for 30 minutes (60 minutes for analogues **6d-e**). The reaction was quenched through addition of 10 mL water and extracted with diethyl ether (3 x 15 mL). The organic phase was washed with brine (15 mL), dried with $MgSO_4$ and the solvent evaporated. Purification by means of column chromatography (SiO_2, petroleum ether/ethyl acetate (60/1), R_f = 0.21) furnished 111 mg pure 6-acetyl-7-hydroxy-2,2-dimethyl-8,8-bis(3-methylbut-2-en-1-yl)-2,8-dihydro-5*H*-chromen-5-one **6a** (0.3 mmol, 56%) as a mixture of two tautomeric forms. Based on comparison with reported data, the major isomer is presumed to be isomer **6a**.[6]

The purification of compound **6c** was not optimized. Compounds **6d-e** were purified by means of reversed phase column chromatography. Complete NMR characterization of derivative **6d** was not possible due to the small amount of pure product obtained. A good indication of the identity and purity of the synthesized product was, however, provided by LC-MS analysis.

6-Acetyl-7-hydroxy-2,2-dimethyl-8,8-bis(3-methylbut-2-en-1-yl)-2,8-dihydro-5*H*-chromen-5-one 6a

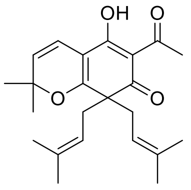

6a

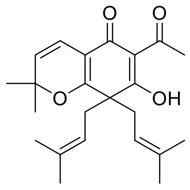

6a'

The ^{1}H NMR spectrum for this compound shows two tautomeric forms **6a** and **6a’** in a 2/1-ratio or vice versa.

^{1}H NMR for the major isomer (400 MHz, $CDCl_3$): δ 1.43 (6H, s); 1.57 (12H, s); 2.46-2.51 (2H, m); 2.58 (3H, s); 2.62-2.69 (2H, m); 4.73-4.80 (2H, m); 5.33 (1H, d, *J* = 10.1 Hz); 6.44 (1H, d, *J* = 10.1 Hz). **^{13}C NMR** for the major isomer (100.6 MHz, $CDCl_3$): δ 18.1 (CH_3); 25.8 (CH_3); 28.0 (CH_3); 28.7 (CH_3); 37.5 (CH_2); 57.0 (C_q); 81.2 (C_q); 106.1 (C_q); 108.4 (C_q); 114.5 (CH); 118.1 (CH); 123.4 (CH); 134.8 (C_q); 172.4 (C_q); 186.0 (C_q); 195.6 (C=O); 199.6 (C=O).

^{1}H NMR for the minor isomer (400 MHz, $CDCl_3$): δ 1.39 (6H, s); 1.57 (12H, s); 2.62-2.69 (4H, m); 2.71 (3H, s); 4.73-4.80 (2H, m); 5.30 (1H, d, *J* = 10.0 Hz); 6.53 (1H, d, *J* = 10.0 Hz). **^{13}C NMR** for the minor isomer (100.6 MHz, $CDCl_3$): δ 18.0 (CH_3); 25.8 (CH_3); 28.5 (CH_3); 28.7 (CH_3); 36.4 (CH_2); 52.5 (C_q); 79.6 (C_q); 111.1 (C_q); 112.2 (C_q); 115.8 (CH); 117.4 (CH); 123.3 (CH); 135.4 (C_q); 164.7 (C_q); 181.5 (C=O); 196.9 (C_q); 202.0 (C=O).

IR (cm^{-1}): $\nu_{C=O}$ = 1654, 1638; ν_{max} = 2976, 2914, 2857, 1524, 1453, 1377, 1358, 1134, 1078, 1101, 876. **MS** (70 eV): m/z (%) = 371 ([M + 1]$^{+}$, 100). Orange oil. Yield after column chromatography: 56%. R_f = 0.21 (SiO_2, PE/EtOAc 60/1).

6-Isovaleryl-5-hydroxy-2,2-dimethyl-8,8-bis(3-methylbut-2-en-1-yl)-2,8-dihydro-7*H*-chromen-7-one 6b

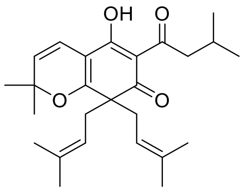

6b

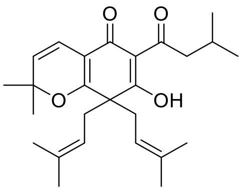

6b'

The ^{1}H NMR spectrum for this compound shows two tautomeric forms **6b** and **6b’** in a 2/1-ratio or vice versa.

^{1}H NMR for the major isomer (400 MHz, $CDCl_3$): δ 0.96 (6H, d, *J* = 6.6 Hz); 1.42 (6H, s); 1.56 (12H, s); 2.07-2.20 (1H, m); 2.45-2.51 (2H, m); 2.65-2.70 (2H, m); 2.91 (2H, d, *J* = 7.0 Hz); 4.77 (2H, t, *J* = 6.9 Hz); 5.33 (1H, d, *J* = 10.0 Hz); 6.46 (1H, d, *J* = 10.0 Hz). **^{13}C NMR** for the major isomer (100.6 MHz, $CDCl_3$): δ 18.1 (2 x CH_3); 22.7 (CH_3); 25.8 (CH_3); 26.0 (CH); 28.7 (CH_3); 37.6 (CH_2); 48.0 (CH_2); 56.9 (C_q); 81.0 (C_q); 106.4 (C_q); 108.3 (C_q); 114.6 (CH); 118.1 (CH); 123.3 (CH) 134.7 (C_q); 172.0 (C_q); 186.6 (C_q); 195.4 (C=O); 202.3 (C=O).

^{1}**H NMR** for the minor isomer (400 MHz, $CDCl_3$): δ 0.96 (6H, d, *J* = 6.6 Hz); 1.39 (6H, s); 1.56 (12H, s); 2.07-2.20 (1H, m); 2.57-2.62 (2H, m); 2.69-2.74 (2H, m); 3.0 (2H, d, *J* = 6.9 Hz); 4.77 (2H, t, *J* = 6.9 Hz); 5.30 (1H, d, *J* = 10.0 Hz); 6.52 (1H, d, *J* = 10.1 Hz). ^{13}**C NMR** for the minor isomer (100.6 MHz, $CDCl_3$): δ 18.0 (CH_3); 22.6 (CH_3); 25.8 (CH_3); 26.1 (CH); 28.6 (CH_3); 36.4 (CH_2); 48.5 (CH_2); 52.6 (C_q); 79.5 (C_q); 111.2 (C_q); 112.3 (C_q); 115.9 (CH); 117.5 (CH); 123.3 (CH); 135.4 (C_q); 164.4 (C_q); 181.4 (C=O); 197.3 (C_q); 204.5 (C=O).

IR (cm^{-1}): $\nu_{C=O}$ = 1655, 1636; ν_{max} = 2959, 2926, 2870, 1518, 1464, 1450, 1383, 1364, 1337, 1196, 1136, 1101, 876. **MS** (70 eV): m/z (%) = 413 ($[M + 1]^{+}$, 100). Yellow oil. Yield after column chromatography: 15%. R_f = 0.20 (SiO_2, PE/EA 60/1).

6-Benzoyl-5-hydroxy-2,2-dimethyl-8,8-bis(3-methylbut-2-en-1-yl)-2,8-dihydro-7*H*-chromen-7-one 6d

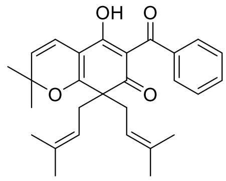

MS (70 eV): m/z (%) 433 (M^{+} + 1, 100).

6-(4-Bromobenzoyl)-5-hydroxy-2,2-dimethyl-8,8-bis(3-methylbut-2-en-1-yl)-2,8-dihydro-7*H*-chromen-7-one 6e

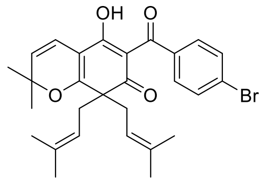

^{1}**H NMR** (400 MHz, $CDCl_3$): δ 1.46 (6H, s); 1.58 and 1.62 (12H, s); 2.48-2.51 (2H, m,); 2.62-2.68 (2H, m); 4.83-4.88 (2H, m); 5.38 (1H, d, *J* = 9.7 Hz); 6.49 (1H, d, *J* = 9.7 Hz); 7.31 (2H, d, *J* = 7.6 Hz); 7.49 (2H, d, *J* = 7.6 Hz). ^{13}**C NMR** (100.6 MHz, $CDCl_3$): δ 18.2 (CH_3); 25.9 (CH_3); 28.7 (CH_3); 37.5 (CH_2); 57.1 (C_q); 81.6 (C_q); 105.9 (C_q); 107.3 (C_q); 114.4 (CH); 118.2 (CH); 123.5 (CH); 125.6 (C_q); 129.6 (CH); 130.8 (CH); 134.9 (C_q); 137.8 (C_q); 173.5 (C_q); 185.0 (C_q); 194.3 (C=O); 195.2 (C=O). **IR** (ATR, cm^{-1}): $\nu_{C=O}$ = 1641; ν_{max} = 1581, 1134, 785, 758, 435, 406. **MS** (70 eV): m/z (%) 511/13 (M^{+} + 1, 100). Yellow crystals. T_m = 120 °C. Yield after automatic reversed phase column chromatography (C18, ACN/H_2O): 10%.

6 ^{1}H and ^{13}C NMR spectra of compounds 4-6

Compound 4e

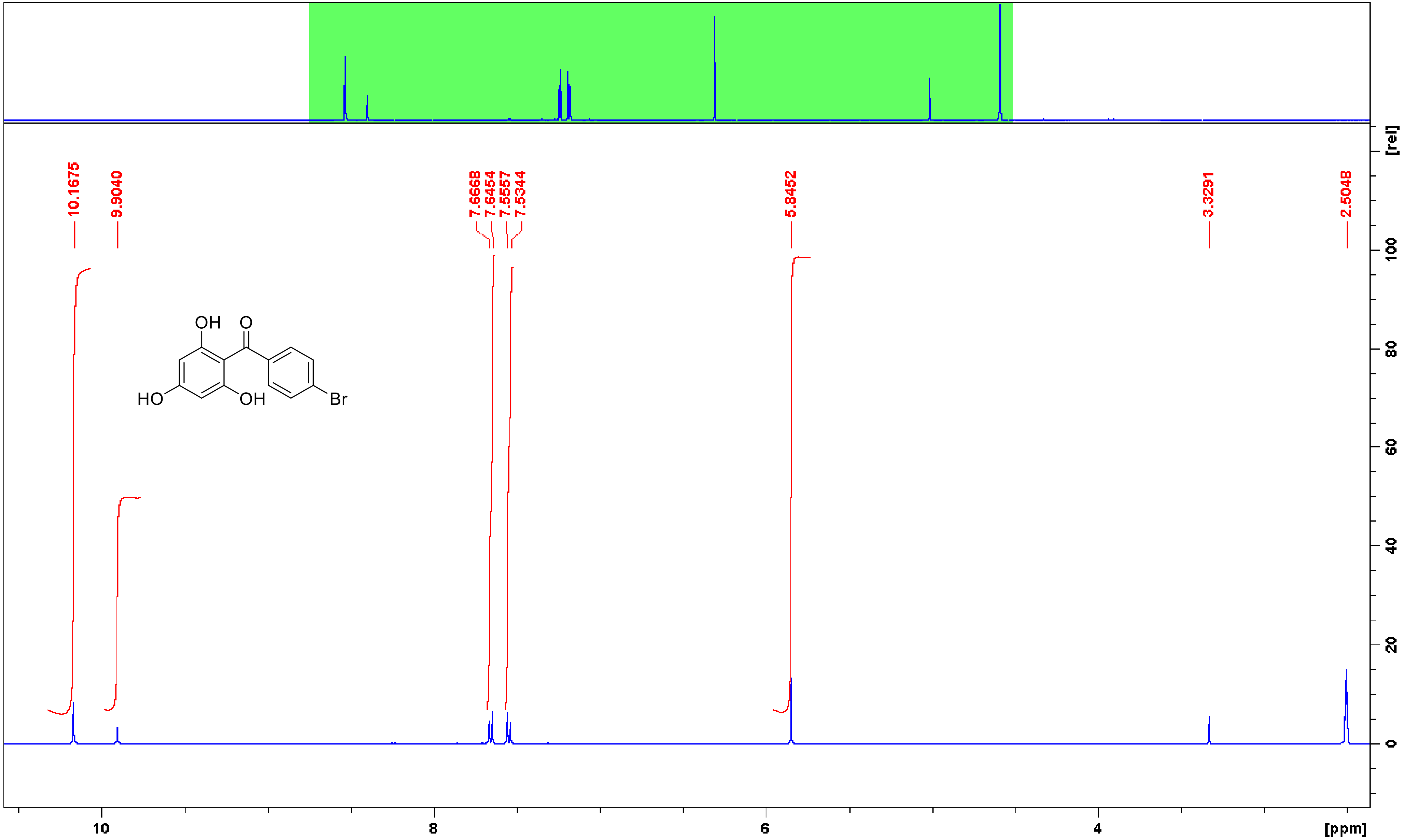

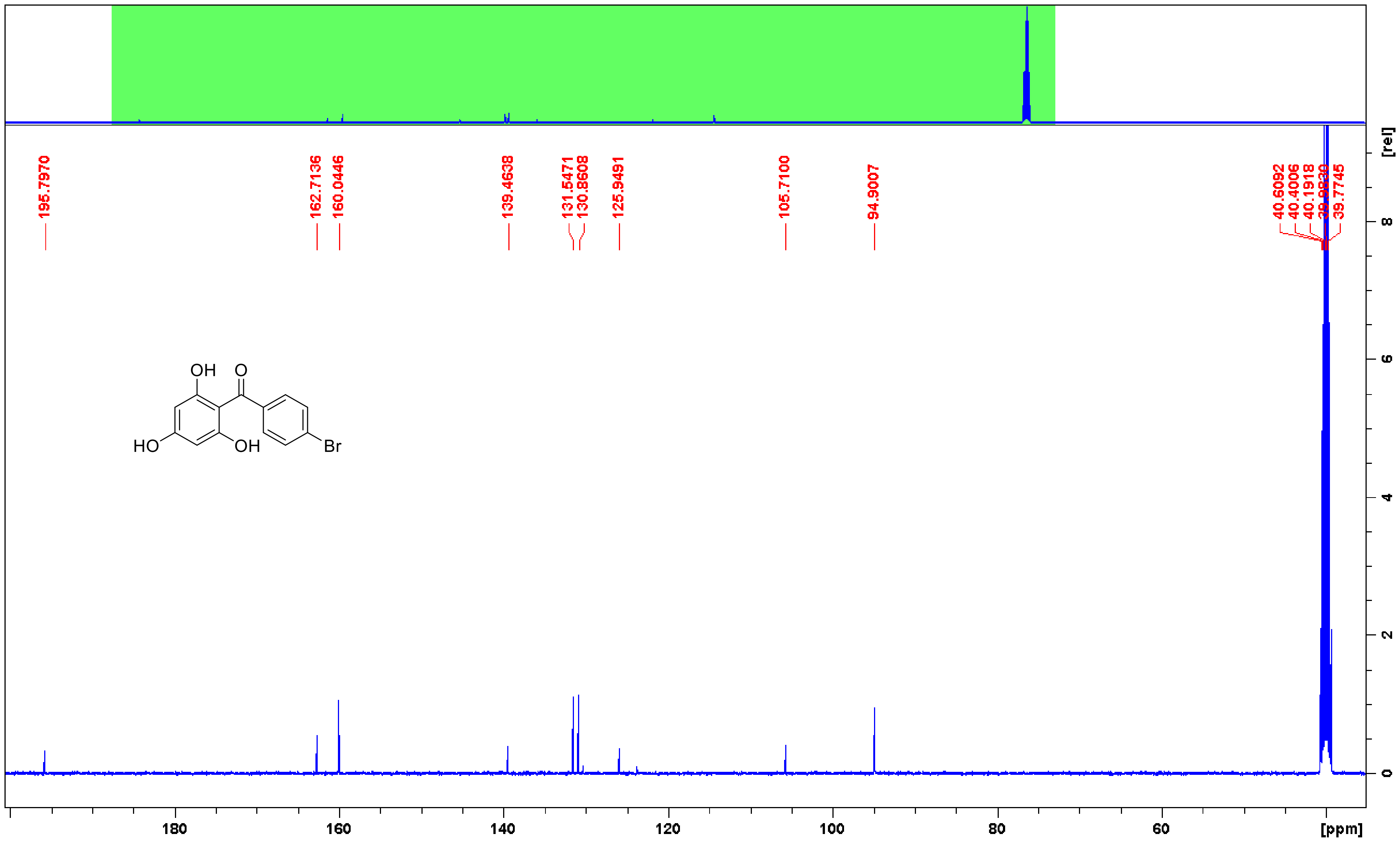

195.7970
162.7136
160.0446
139.4638
131.5471
130.8608
125.9491
105.7100
94.9007
40.6092
40.4006
40.1918
39.9830
39.7745
OH
O
HO
OH
Br
[rel]
[ppm]
180
160
140
120
100
80
60
0
2
4
6
8

Compound 6a, 6a'

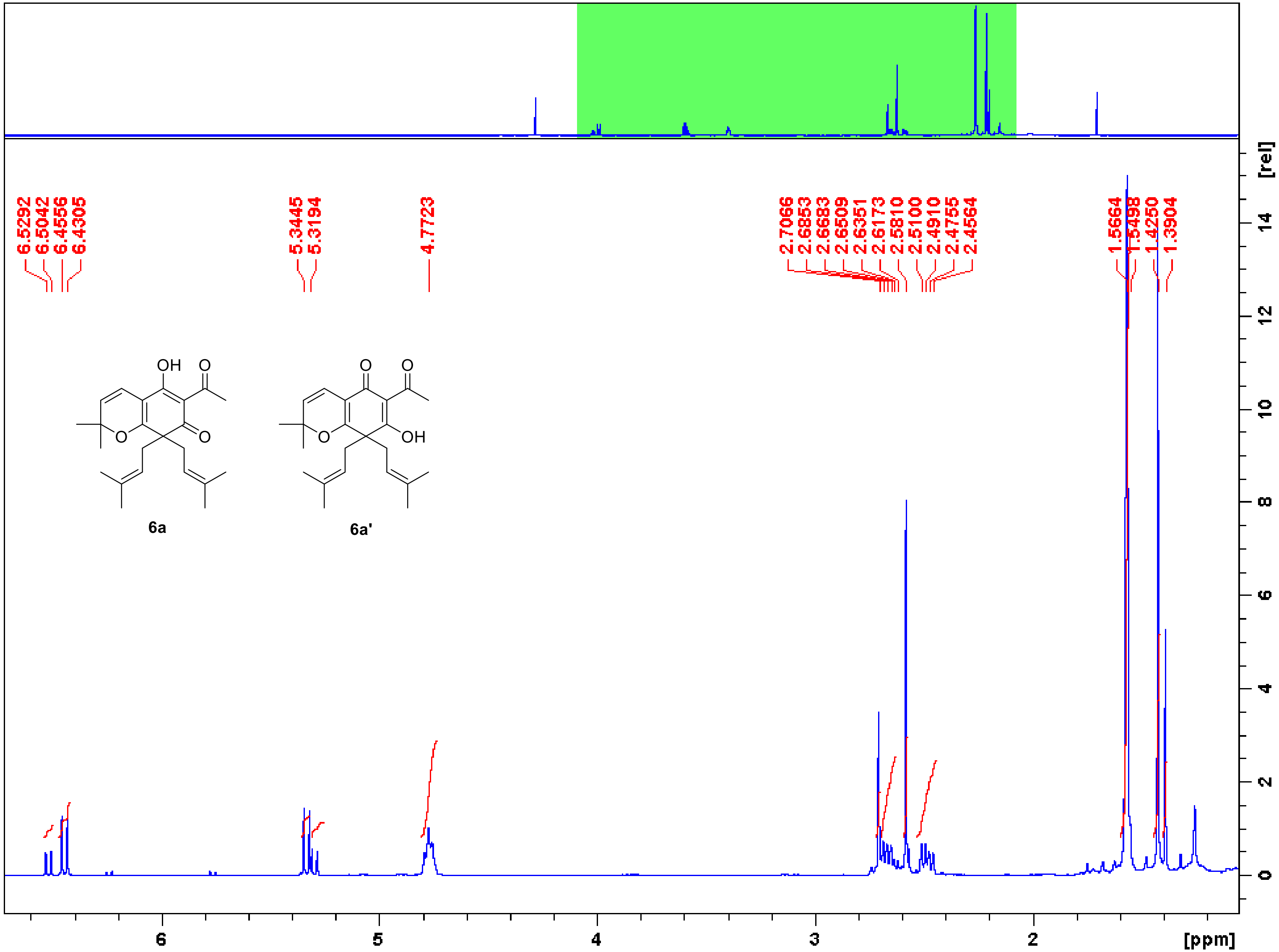

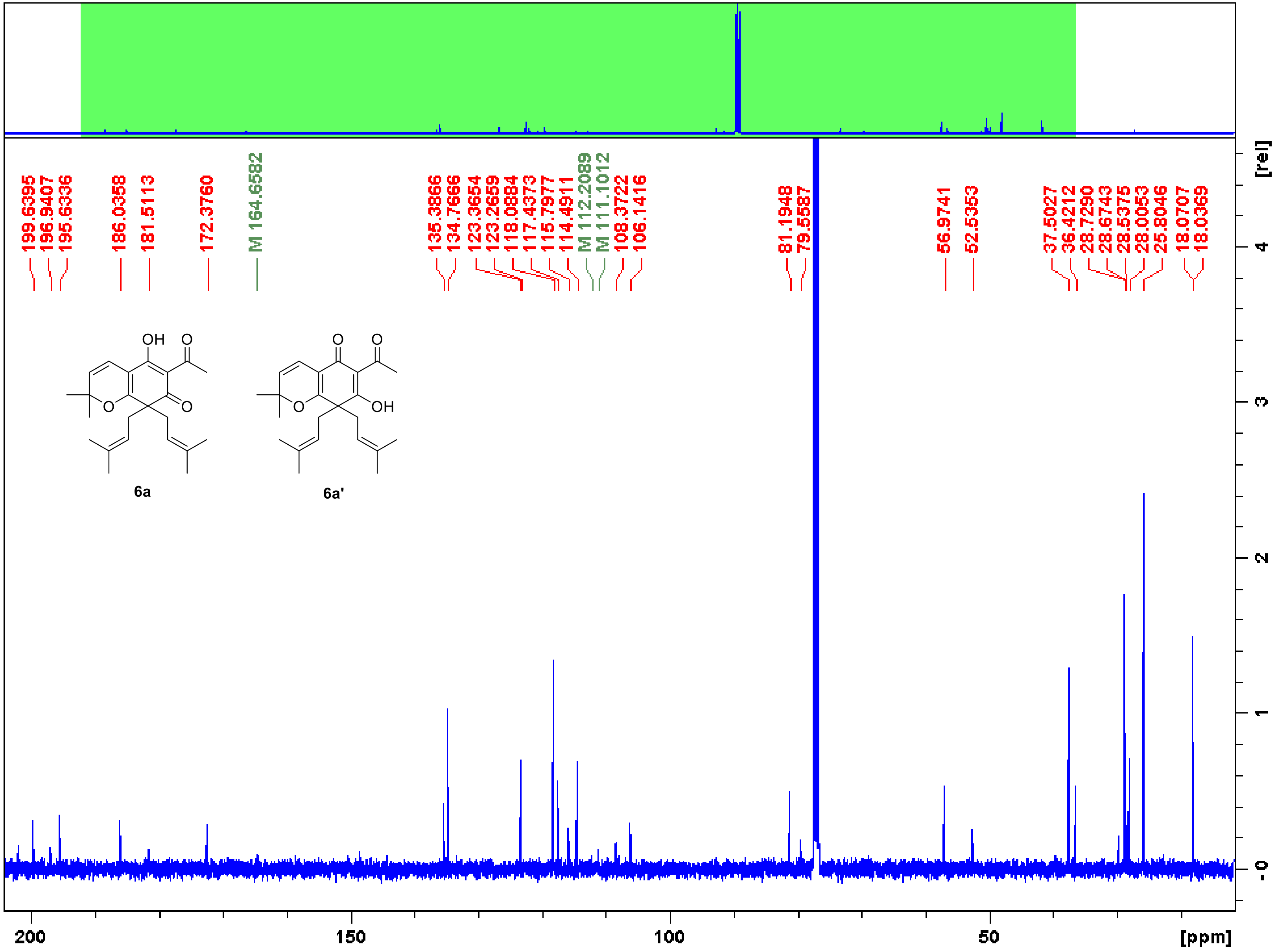
199.6395
196.9407
195.6336
186.0358
181.5113
172.3760
M 164.6582
135.3866
134.7666
123.3654
123.2659
118.0884
117.4373
115.7977
114.4911
M 112.2089
M 111.1012
108.3722
106.1416
81.1948
79.5587
56.9741
52.5353
37.5027
36.4212
28.7290
28.6743
28.5375
28.0053
25.8046
18.0707
18.0369
OH
O
O
OH
6a
6a'
[rel]
200
150
100
50
[ppm]
4
3
2
1
0

Compound 6b, 6b'

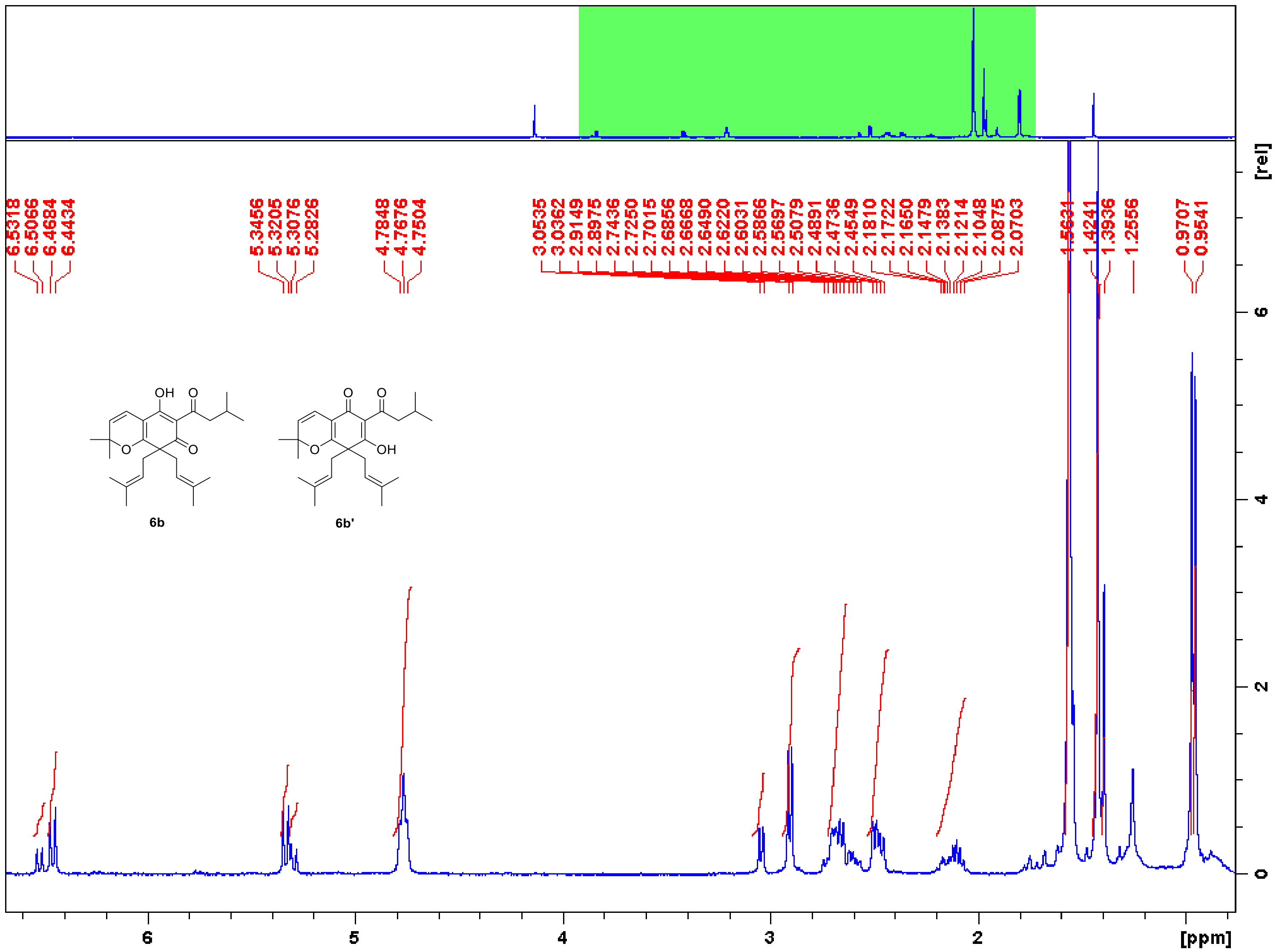

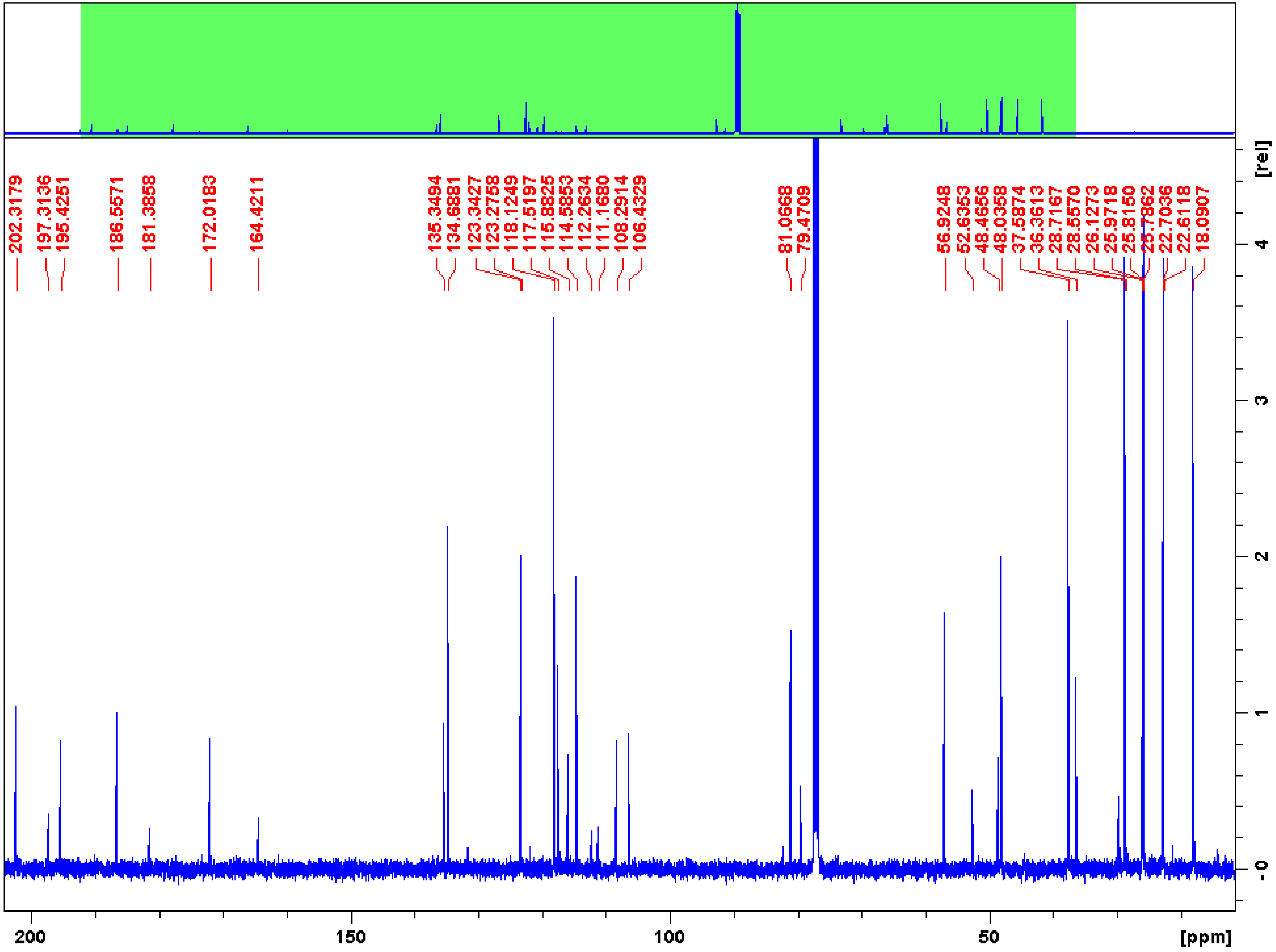
202.3179
197.3136
195.4251
186.5571
181.3858
172.0183
164.4211
135.3494
134.6881
123.3427
123.2758
118.1249
117.5197
115.8825
114.5853
112.2634
111.1680
108.2914
106.4329
81.0668
79.4709
56.9248
52.6353
48.4656
48.0358
37.5874
36.3613
28.7167
28.5570
26.1273
25.9718
25.8150
25.7862
22.7036
22.6118
18.0907
[rel]
4
3
2
1
-0
200
150
100
50
[ppm]

Compound 6e

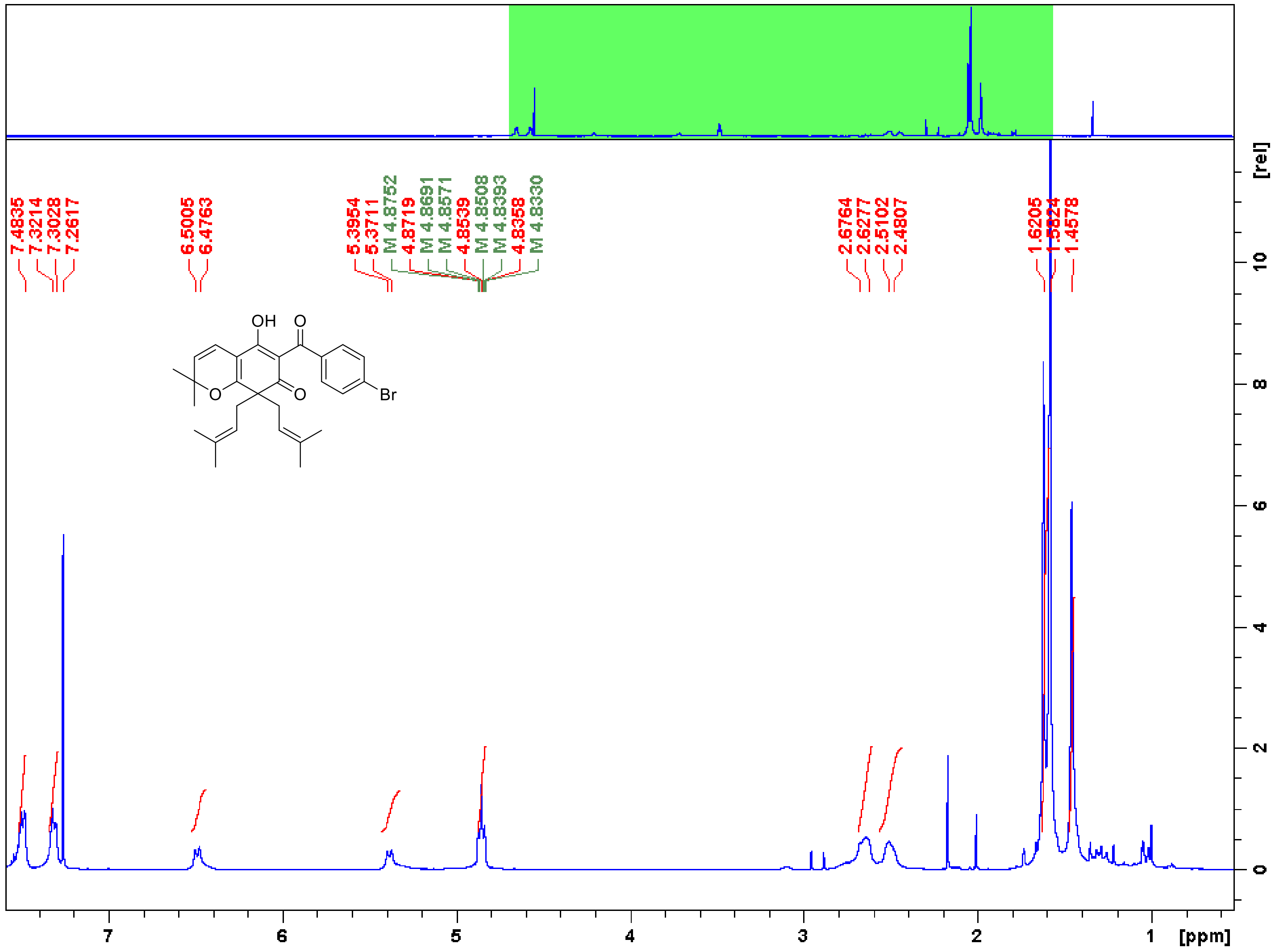
7.4835
7.3214
7.3028
7.2617
6.5005
6.4763
5.3954
5.3711
M 4.8752
4.8719
M 4.8691
M 4.8571
4.8539
M 4.8508
M 4.8393
4.8358
M 4.8330
2.6764
2.6277
2.5102
2.4807
1.6205
1.5824
1.4578
OH
O
O
O
Br
[rel]
[ppm]

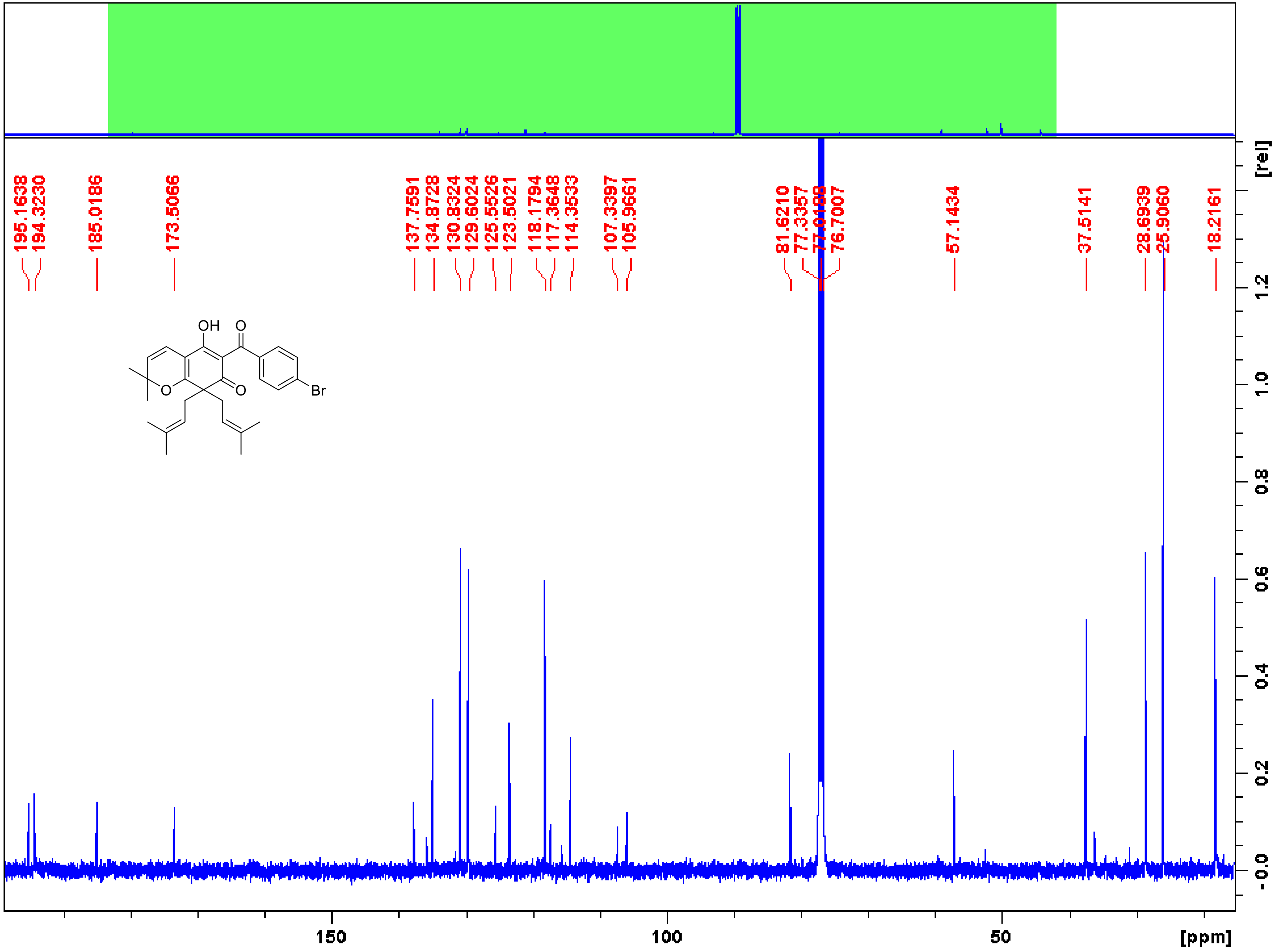
195.1638
194.3230
185.0186
173.5066
137.7591
134.8728
130.8324
129.6024
125.5526
123.5021
118.1794
117.3648
114.3533
107.3397
105.9661
81.6210
77.3357
77.0188
76.7007
57.1434
37.5141
28.6939
25.9060
18.2161
OH
O
O
O
Br
[rel]
1.2
1.0
0.8
0.6
0.4
0.2
-0.0
150
100
50
[ppm]

REFERENCES

[1] M. Morkunas, L. Dube, F. Götz, M. E. Maier, *Tetrahedron* **2013**, *69*, 8559.

[2] J. W. van Klink, J. J. Brophy, N. B. Perry, R. T. Weavers, *J. Nat. Prod.* **1999**, *62*, 487.

[3] G. P. Mrug, S. P. Bondarenko, V. P. Khilya, M. S. Frasinyuk, *Chem. Nat. Compd.* **2013**, *49*, 235.

[4] M. L. Tucknott, *The chemical synthesis of natural and novel β-acid derivatives for biological evaluation as anticancer and antibacterial agents*, PhD thesis, *Kingston University* **2013**, 10-21.

[5] M. Collins, D. R. J. Laws, J. D. McGuinness, J. A. Elvidge, *J. Chem. Soc. C* **1971**, 3814.

[6] J. H. George, M. D. Hesse, J. E. Baldwin, R. M. Adlington, *Org. Lett.* **2010**, *12*, 3532.
